# Diversity of Cultivable Methane-Oxidizing Bacteria in Microsites of a Rice Paddy Field: Investigation by Cultivation Method and Fluorescence *in situ* Hybridization (FISH)

**DOI:** 10.1264/jsme2.ME11327

**Published:** 2012-03-23

**Authors:** Dayéri Dianou, Chihoko Ueno, Takuya Ogiso, Makoto Kimura, Susumu Asakawa

**Affiliations:** 1 Centre National de la recherche Scientifique et technologique, 03 BP 7192 Ouagadougou, Burkina Faso; 2 Graduate School of Bioagricultural Sciences, Nagoya University, Furocho, Chikusa, Nagoya 464–8601 Japan

**Keywords:** Cultivable bacteria, diversity, methane-oxidizing bacteria, FISH, rice paddy field microsite

## Abstract

The diversity of cultivable methane-oxidizing bacteria (MOB) in the rice paddy field ecosystem was investigated by combined culture-dependent and fluorescence *in situ* hybridization (FISH) techniques. Seven microsites of a Japanese rice paddy field were the focus of the study: floodwater, surface soil, bulk soil, rhizosphere soil, root, basal stem of rice plant, and rice stumps of previous harvest. Based on *pmoA* gene analysis and transmission electron microscopy (TEM), four type I, and nine type II MOB isolates were obtained from the highest dilution series of enrichment cultures. The type I MOB isolates included a novel species in the genus *Methylomonas* from floodwater and this is the first type I MOB strain isolated from floodwater of a rice paddy field. In the type I MOB, two isolates from stumps were closely related to *Methylomonas* spp.; one isolate obtained from rhizosphere soil was most related to *Methyloccocus-Methylocaldum-Methylogaea* clade. Almost all the type II MOB isolates were related to *Methylocystis* methanotrophs. FISH confirmed the presence of both types I and II MOB in all the microsites and in the related enrichment cultures. The study reported, for the first time, the diversity of cultivable methanotrophs including a novel species of type I MOB in rice paddy field compartments. Refining growth media and culture conditions, in combination with molecular approaches, will allow us to broaden our knowledge on the MOB community in the rice paddy field ecosystem and consequently to implement strategies for mitigating CH_4_ emission from this ecosystem.

Methane (CH_4_) is an important greenhouse gas ranking second to carbon dioxide, and wetland rice fields are one of the major sources of methane emission, accounting to 5–19% of the global CH_4_ budget (15). Methane emission from a rice field is the net effect of its production (methanogenesis) and its oxidation (methanotrophy). About 60–90% of the produced CH_4_ is oxidized *in situ* before it escapes to the atmosphere (56). Microbial CH_4_ oxidation driven by methane-oxidizing bacteria (MOB) is the only biological suppression of methane emission from rice fields, and consequently MOB are considered to be important regulators of methane effluxes from this ecosystem (39). MOB are a unique group of bacteria that oxidize CH_4_ with molecular O_2_ and use it as a carbon and energy source (9, 26). It is generally recognized that CH_4_ oxidation occurs at oxic-anoxic interfaces in rice paddy fields, at the soil-water interface, and in the rhizosphere and rhizoplane of rice plants with available O_2_ and CH_4_(18, 21). Consequently, intermittent water management (flooding/draining) and the resulting significant biogeochemical processes generated (34) may affect CH_4_ biological oxidation and probably the composition of MOB in the microsites of the rice paddy field ecosystem.

Methanotrophs are mostly classified into the *Gammaproteo-bacteria* (type I MOB) and the *Alphaproteobacteria* (type II MOB) based on their intracytoplasmic membrane structure, carbon-assimilation pathway, phospholipid fatty acid profile, and phylogenetic placement. Both types I and II MOB have been found in rice field bulk soil, rice rhizosphere, soil-water-interface, and on rice roots using phospholipid fatty acid analysis (38, 50), PLFA-stable isotope probing (50), 16S rDNA and *pmoA* sequencing (18, 20, 21, 32, 35, 40, 50). In contrast with these results revealed through molecular approaches and despite the heterogeneity of microsites in the rice paddy field ecosystem with regard to O_2_, CH_4_, and nutrient availability, only type II MOB have been isolated from rice paddy soil and rice roots using cultivation methods (16, 24, 52, 53). However more recently, using a combined molecular and cultivation technique, a mesophilic type I MOB was isolated at the soil-water-interface from a rice paddy field in Uruguay (21, 23), and is the first and sole type I strain isolated from rice paddy fields. Therefore, cultivable MOB diversity associated with microsites in rice paddy field ecosystem remains to be elucidated. In particular, no type I MOB have been isolated from the rice rhizosphere, rice roots, bulk soil or floodwater, although their presence was revealed using molecular techniques as mentioned above. It is commonly accepted that only a small fraction of microbes is cultivable and that molecular approaches always cover a broader spectrum of microbial diversity than cultivation methods (3, 22, 47), although in some cases this seems untrue (17, 36, 45). Therefore, in line with Hengstmann *et al.*(28), Oremland *et al.*(45) and Donachie *et al.*(17), implementing comprehensive strategies, which include combined improvement of culturing and molecular techniques, may help to gain more insight into cultivable MOB diversity in the rice paddy field ecosystem.

In order to address this problem, we used cultivation techniques and fluorescence *in situ* hybridization (FISH) in a combined approach to characterize the cultivable MOB inhabiting floodwater, surface soil, rice stumps from the previous harvest, bulk soil, rhizosphere soil, root, and rice stem from a Japanese rice paddy field.

## Materials and Methods

### Field site

The studied site was a rice-wheat double cropping paddy field in Aichi-ken Anjo Research and Extension Center, central Japan (latitude 34°48′N, longitude 137°30′E). Principal soil characteristics as described by Watanabe *et al.*(57) and Jia *et al.*(32) were as follows: total C, 12.8 g kg^−1^; total N, 1.1 g kg^−1^; pH (H_2_O), 6.3; amorphous Fe content, 3.76 g kg^−1^. The soil was classified as Oxyaquic Dystrudept with a clay content of 230 g kg^−1^. The study was conducted during the summer season in 2003 and 2008. Two Japonica-rice varieties (*Matsuribare* and *Aichinokaori SBL*) were cultivated in the paddy field in 2003 (plot E2) and 2008 (plot B4) in the paddy field, respectively.

### Sampling scheme and rice paddy field microsites

Samples were taken at the tillering stage (August 4 and 11, 2003), at the flowering stage (September 4, 2003), and at the maturity and harvest stage (October 10, 2008) from plots E2 (in 2003) and B4 (in 2008), respectively. The samples in 2003 were used for isolation of MOB and the samples in 2008 for FISH observation of MOB by extraction of cells or after enrichment. We focused on seven rice paddy field compartments (microsites; abbreviations in parentheses) for this study: floodwater (Fw), surface soil (SS), bulk soil (S), rhizosphere soil (RS), total root (homogenate) (Rt), basal stem (Ste), and stump (Stu; only in 2003) of the previous harvest. Floodwater, soil surface, and bulk soil (soil) between rice plants footpaths, and stumps were collected at the field. All samples were collected from three points randomly distributed in the plot and then mixed. Floodwater (5–10 cm depth) was collected with a 100 mL plastic beaker and poured into sterile plastic bottles. Surface soil (0–0.5 cm) and bulk soil (2–10 cm) were collected with a small trowel and plastic bags. Samples from the other microsites of the rice field were obtained after taking the total rice plant with soil (three cores of approximately 20 cm diameter by 18 cm depth randomly distributed in the plot) to the laboratory. From each core, a rice plant was dug out by hand and shaken to remove loose soil on the roots. The fine layer of soil firmly attached to the roots was released into 100 mL nitrate mineral salt (NMS) medium. The resulting mixed soil solution obtained from the three cores (approximately 1 g [10 mL]^−1^) was referred to as rhizosphere soil in this study. After removing the rhizosphere soil, the roots were rinsed again repeatedly with sterile distilled water until the water ran clear, before removing the remaining water by pressing between paper. The roots were then cut into small pieces and referred to as roots in this study. Basal stems (0–2 cm) of rice plants and stumps were rinsed with sterile distilled water to remove the remaining soil before they were pressed between paper and cut into small pieces as for roots. Bulk soil and surface soil (10 g) were ground with a sterile mortar and pestle to give a homogenous mixture. The resulting samples were used for enrichment and isolation of MOB.

### Preparation of enrichment cultures and MOB isolation

All enrichment cultures were prepared using NMS medium (59). Trace elements were added before autoclaving according to Roslev and King (49) to give the following final concentrations: zinc chloride, 2.0 μM; cupric chloride, 2.0 μM; sodium bromide, 1.0 μM; sodium molybdate, 0.5 μM; manganese chloride, 2.0 μM; potassium iodide, 1.0 μM; boric acid, 2.0 μM; cobalt chloride, 1.0 μM; nickel chloride, 1.0 μM; and iron sulphate, 40.0 μM. The pH of the medium was then adjusted to 6.8 by addition of 1 M NaOH. The medium was then autoclaved at 121°C for 20 min.

MOB were isolated from the 2003 samples without enrichment (A), and from enrichment cultures prepared according to Whittenbury *et al.*(59) (B) and Bowman *et al.*(10) (C). Some factors (CH_4_ pressure, shaking culture) were reported to affect the composition of MOB populations (4, 25, 27, 31, 60); therefore, we performed enrichments under 5 and 25% (v/v) CH_4_ phase (D), and under 20% (v/v) CH_4_ phase with shaking (E). For enrichment B (59), samples (0.5 g) except floodwater were added to 10 mL sterile NMS liquid medium in 60 mL flasks before the flasks were sealed with butyl rubber stoppers and an aluminium seal. Methane (6 mL) was then passed through a 0.20 μm pore filter with a gas-tight syringe and injected into each flask, giving about 10% (v/v) CH_4_ in the head gas-phase. The flasks were incubated statically at 30°C in the dark for 3–4 weeks. Enrichment from floodwater was performed similarly with 5 mL sample added to 5 mL autoclaved NMS medium. Enrichment C according to Bowman *et al.*(10) was prepared as follow: Surface soil, soil, root, stem, and stump materials (1 g) were added to 15 mL sterile NMS liquid medium in 34 mL test tubes (18 mm i.d.×180 mm) with glass beads and mixed for 3–5 min. Rhizosphere soil solution with the same concentration as the material was treated similarly. The supernatants of the solutions and floodwater (250 mL) were filtered through 0.20 μm pore filters. The filters were placed into 60 mL flasks containing 10 mL sterile NMS liquid medium. The flasks were then incubated statically at 30°C and 37°C under about 20% (v/v) CH_4_ for 3–4 weeks. At the end of the incubation period, pellicles formed at the surface and solution were used separately for serial dilutions and isolation. In enrichments under 5 and 25% (v/v) CH_4_ (D), floodwater and rhizosphere soil (60 mL), and 60 mL of 5% NMS solutions (w/v) from surface soil, soil, root, stem, and stump samples were placed in sterile 120 mL flasks. The flasks were incubated under approximately 5% and 25% (v/v) CH_4_ statically at 30°C for 3–4 weeks. For the enrichment E series, samples (200 or 320 mL) used for enrichment D were placed in 500 and 700 mL flasks. The flasks were incubated under about 20% CH_4_ at 30°C with shaking (150 rpm) for 3–4 weeks.

In 2008, enrichments for FISH observation were performed according to Bowman *et al.*(10) (C), Wise *et al.*(60) and Auman *et al.*(5) with respect to type I MOB preferential cultivation. Surface soil, soil, rhizosphere soil, root, and stem (1 g) were mixed in 9 mL NMS medium with glass beads by vortexing vigorously for 3–5 min. Floodwater (250 mL) was centrifuged at 13,000 rpm for 10 min at 4°C before the pellet was resuspended in 9 mL NMS with glass beads by vortexing as above. The obtained suspensions were serially 10-fold diluted (10^−1^ to 10^−10^) in 18 mL NMS medium supplemented or not with 20 μM copper in 121 mL flasks and the headspace was replaced by air containing 10% CH_4_+2% CO_2_ (v/v) or 45% CH_4_+5% CO_2_ (v/v). The flasks were then incubated at 30°C in the dark under moderate shaking (150 rpm) for 3–4 weeks. Additional enrichment series under 20% (v/v) CH_4_ were performed in parallel according to method E described above.

Growth turbidity and CH_4_ uptake in the flasks were periodically checked with a gas chromatograph (GC-9; Shimadzu, Kyoto, Japan), using a Porapak N column connected to a flame ionization detector (FID). The oven temperature was 60°C and He served as carrier gas.

### MOB isolation in pure culture

For isolating MOB in 2003, the enrichment cultures were diluted by serial 10-fold dilution in 9 mL NMS medium in 34 mL test tubes up to 10^−9^ (2 dilution series). From the C enrichment process (10), pellicles formed on the surface of filters and the culture solution were treated separately. The 10^−3^ to 10^−9^ series were then spread onto NMS agar (7 mL) slopes in 34 mL test tubes. The tops of the tubes were closed with butyl rubber stoppers and CH_4_ (6 mL) was injected into each tube, giving about 18% (v/v) CH_4_ in the head gas-phase. Control tubes without CH_4_ injection were prepared in duplicate to detect colonies of non-methane-oxidizing contaminants. The tubes were then incubated at 30°C in the dark and observed at 3-day or 1-week intervals over 3–4 weeks. Single colonies formed on the NMS agar slopes were transferred repeatedly onto fresh NMS agar slopes and incubated again for 1–3 weeks. Then, cultures were checked for CH_4_ utilization by analyzing the head gas-phase using a GC (GC-9; Shimadzu, Kyoto, Japan). Single colonies from positive cultures regarding CH_4_ utilization were then transferred into NMS liquid medium (9 mL) and incubated in a slanted position with moderate shaking (156 rpm) at 30°C for 5–10 days. Positive cultures (0.5–1 mL) were serially diluted (10^−1^ to 10^−10^) in NMS liquid medium and then incubated for the same period under the same conditions. The highest dilutions series showing good and stable CH_4_ utilization after three subsequent transfers to liquid NMS medium were then purified again on NMS agar slopes by successive transfers of a single colony and a 2–3-week incubation period at 30°C.

Isolates were considered to be pure if colonies were similar morphologically. The purity of the cultures was then ascertained by microscopic examination under phase-contrast and by checking growth on NMS agar under a CH_4_ gas-phase or not. The presence of non-MOB contaminants was checked by examining growth on NMS agar medium supplemented with 0.1% (w/v) sucrose or not after incubating without CH_4_ in the gas-phase at 30°C for 7 days.

### Morphological and physiological traits of the isolates

Colony morphology and pigmentation were determined using 7–10-day-old NMS slant cultures. Cell morphology of 5–7-day- and over 3-week-old cultures (on agar slant or liquid culture) was examined by phase-contrast microscopy using wet mounts with an Olympus model BX50 microscope. The cell intracytoplasmic membrane structure was observed by transmission electron microscopy (TEM) with ultra-thin sections of cells fixed with 3% (v/v) glutaraldehyde and 1% (w/v) osmium tetroxide and stained with 2% (w/v) uranyl acetate and lead stain solution (Sigma-Aldrich) using a Hitachi H-7500AMT Advantage HR transmission electron microscope. Cyst formation was tested as described by Vela and Wyss (54). Exospore formation was screened by determining the viability of 3-week-old cultures after heating at 80°C for 20 min using a water bath (10), then checking for growth in NMS medium. Growth of isolates at 37°C and 45°C in NMS liquid medium under an 18% (v/v) methane gas-phase was determined. The ability of isolates to use methanol as a carbon source at a concentration of 0.1% (w/v) was tested after 5–10-day incubation at 30°C, according to Whittenbury *et al.*(59) and Bowman *et al.*(11). Gram staining was performed on 1–2-week-old cultures by Hucker’s modification method.

### Phylogenetic analysis of 16S rDNA and *pmoA* sequences

Cells of isolates were suspended in 600 μL TESS buffer (25 mM Tris-HCl; 5 mM EDTA 2Na; 50 mM NaCl; 25% [w/v] sucrose) with lysozyme (5 mg mL^−1^), placed on ice for 30 min, and 30 μL of 10% (w/v) SDS and 20 μL Proteinase K (10 mg mL^−1^) were added. The preparations were incubated at 50°C for 2 hours and then DNA was extracted with phenol-chloroform-isoamylalcohol and chloroform-isoamylalcohol reagents and by isopropanol and ethanol precipitation. Gene fragments of 16S rRNA and *pmoA* were amplified by PCR using the following primers: 27f/1492r (58) and A189f/A682r (30) or mb661r (14), respectively. The sequences were determined with a 373S DNA Automated Sequencer or genetic analyzers (PRISM 310 Genetic Analyzer, PRISM Genetic Analyzer 3100 and ABI 3130 Genetic Analyzer; Applied Biosystems) with a DYEnamic ET Terminator Cycle Sequencing Kit (Amersham Pharmacia Biosciences, CA, USA) or Big Dye Terminator v3.1 Cycle Sequencing Kit (Applied Biosystems). The 16S rRNA gene and the deduced amino acid sequences of the *pmoA* were subjected to the BLAST program in the DNA Data Bank of Japan (DDBJ; http://www.ddbj.nig.ac.jp/Welcome-j.html) to search for related sequences. Pairwise similarity values of 16S rRNA gene sequences were calculated with a global alignment algorithm using the EzTaxon server (http://www.eztaxon.org/; [13]). Phylogenetic trees were constructed using the neighbor-joining method with the ClustalW program based on the web site of the DNA Data Bank of Japan (DDBJ; http://www.ddbj.nig.ac.jp/Welcome-j.html) by 1,000 replication bootstrap analysis and nj plot software (46).

### FISH from MOB isolates and enrichment cultures

Type I-specific probes Mγ84 (3′-AGCCCGCGACTGCTCACC-5′) and Mγ705 (3′-CTAGACTTCCTTGTGGTC-5′), and type II-specific probe Mα450 (3′-CTATTACTGCCATGGACCTA-5′), which allow the detection of type I and type II methanotrophs in cell mixtures as well as in natural samples (19), were used in the study. The domain-specific probe EUB338 (3′-GCTGCCTCCCG TAGGAGT-5′) was used as a positive control to test the efficiency of hybridization (2). All probes were labeled with Cy3 for Mγ84, Mγ705, Mα450 and EUB 338. For probe Mα450 targeting type II methanotrophs, *Methylobacter luteus* (type I) was used as a negative control to check the specificity of hybridization, and for probes Mγ84 and Mγ705, *Methylocystis* sp. Rp1 (type II) was used (24). Prior to applying FISH for the isolated cells, a formamide gradient (between 0% and 80% [v/v]) in the hybridization buffer was screened to assess the optimal stringency and specificity for probes Mγ705, Mγ84, Mα450, and EUB338, and 20% (v/v) was selected in agreement with Eller *et al.*(19) for the study.

The fixation procedure was adapted from Eller *et al.*(19). Five-day-old cultures (exponential growth phase) of MOB isolates (1 mL) or enrichment culture (1.5–2 mL) were harvested by centrifugation (15,000 rpm, 10 min, 4°C). Cell pellets were resuspended in 100 μL phosphate-buffered saline (PBS, pH 7.0) prior to the addition of 300 μL of freshly prepared 4% (w/v) paraformaldehyde (in PBS). Samples were then left for 2 h at room temperature for fixation before they were washed five times with 500–1,000 μL PBS. Pellets were then resuspended in ethanol/PBS as 1:1 (v/v) and stored at − 20°C until hybridization.

The protocol of whole cell hybridization was adapted from Amann *et al.*(2), Bourne *et al.*(8) and Eller *et al.*(19). Hybridizations were performed in 8-well Teflon-coated slides. Slides were precleaned by soaking for 1 h in 99.5% ethanol and rinsing in distilled water before they were washed in 1% (v/v) HCl and 70% (v/v) ethanol, and then air-dried. Fixed cell suspension (1–1.5 μL) was transferred to each well. The slides were dried at 46°C for 10 min in an oven (HB-80; TAITEC, Koshigaya, Japan), subsequently dehydrated by immersing in 50, 80, and 99.5% (v/v) aqueous ethanol for 3 min each, and then air-dried, before wells were covered with 8 μL hybridization buffer (Tris 2.4 g L^−1^, SDS 2.0 g L^−1^, EDTA 2.0 g L^−1^, NaCl 0.9 M, 20% [v/v] formamide, pH 7.4). To each well, 1 μL probe solution (50 ng μL^−1^) was added and hybridization was carried out for 1 h 30 min (cultures) or 2 h (cells extracted from microsite samples) at 46°C in a water-saturated atmosphere chamber (50 mL Falcon tube containing a piece of a 150 mm 5C-filter paper soaked in 4 mL hybridization buffer) in a hybridization incubator (HB-80; TAITEC). Unbound nucleotides were removed by rinsing the slides with 20 mL washing buffer (Tris 2.4 g L^−1^, SDS 2.0 g L^−1^, EDTA 2.0 g L^−1^, pH 7.4, 225 mM NaCl corresponding to 20% [v/v] formamide concentration in hybridization buffer) and prewarmed to 48°C in a water bath. Subsequently, the slides were incubated with 8 μL washing buffer per well for 20 min at 48°C in a water-saturated atmosphere chamber (50 mL Falcon tube containing a piece of a 150 mm 5C-filter paper soaked in 4 mL washing buffer) in the hybridization incubator. They were then rinsed again with 20 mL washing buffer prewarmed to 48°C, air-dried, and then DNA-stained with 10 μL of 50 μL mL^−1^ DAPI (4′,6-diamino-2-phenylindole) solution for 10–15 min at room temperature in the dark. The remaining DAPI solution was removed by rinsing the slides with distilled water.

After air drying, slides were mounted in immersion oil (05; Olympus). Epifluorescence microscopy was performed with an Olympus BX-FLA microscope (Olympus) equipped with a 100×/1.25 Oil Ph3 immersion lens and fitted with a 50-W high pressure bulb and an image recorder (iCY-SHOT DXC-S500; Sony, Japan). The following Olympus light filter sets were used: NV (U-MNV) for phase contrast, WIG (U-MWIG) for Cy3 and NUA (U-MNUA) for DAPI.

### Detection of MOB inhabiting rice paddy field microsites by FISH

Bacterial cells were extracted from the rice paddy field microsites using a method adapted from Eller *et al.*(19). Washed roots, surface soil, bulk soil, rhizosphere soil, basal stem (10–11 g), and floodwater (250 mL) were shaken with glass beads (diameter 1 mm) and sterile distilled water (30 mL in 50 mL Falcon tubes) on a horizontal shaker (TS-4N; TAITEC) for 30 min at 200 rpm. Decantation was then performed on ice for 15 min before transferring the supernatant into 15 mL sterile Falcon tubes. The extraction process was repeated and then the resulting mixed supernatants for each sample were centrifuged at 500–1,000 rpm for 2 min at 4°C to precipitate large particles. The resulting supernatants were centrifuged at 12,000–15,000 rpm for 10 min at 4°C and then the pellet (cells) was resuspended in 100 μL (or more according to pellet amount) PBS at pH 7.0 before adding 300 μL of 4% paraformaldehyde (w/v in PBS). After gentle mixing, cells were then left to fix for 2–16 h at room temperature. Whole cell hybridization and microscopy were then performed as described above for pure culture. FISH was performed in parallel on the same microsite samples collected on October 10, 2008 and on the issued enrichment cultures with appreciable CH_4_ uptake and turbidity.

### Accession numbers

The GenBank/EMBL/DDBJ accession numbers for the 16S rRNA gene and *pmoA* sequences of MOB strains in the present study are AB669143–AB669172.

## Results

### Characteristics of MOB isolates

**Fig. 1:**
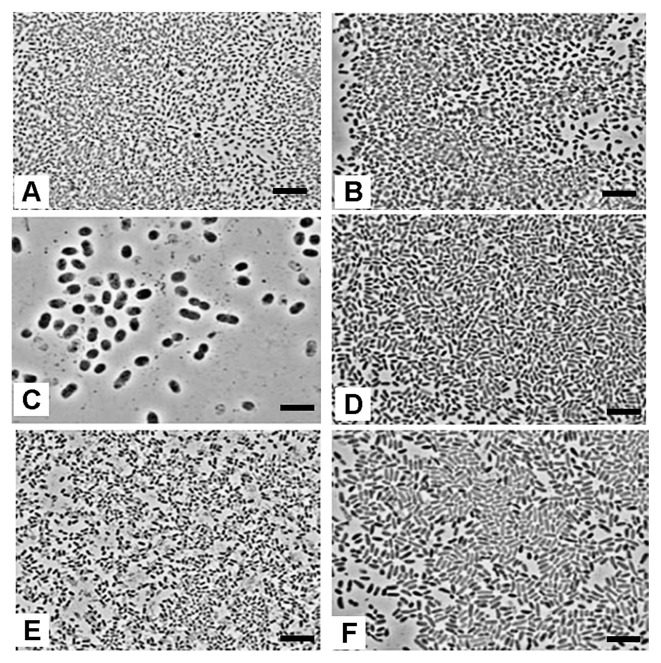
Phase-contrast photomicrographs of methane-oxidizing bacterial isolates from rice paddy field microsites. (A) Fw12E-Y, (B) SS10D-Y-Pr, (C) RS11D-Pr, (D) S18C-Re, (E) Stu1B-Pr, and (F) Ste3C-Re. Bar=5 μm.

From the enrichment cultures, 13 MOB isolates were obtained from floodwater (two), surface soil (two), bulk soil (two), rhizosphere soil (three), stem (one), and stump (three) (Table 1). The name of isolates was designated with the source, isolate number, enrichment method, and pigment, *e.g.* Fw12E-Y. All the MOB isolates were motile and stained Gram negative. Exospore or cyst formation was not observed for any MOB isolates. Cell morphology under phase-contrast microscopy varied from rod, short rod, short rod-curve, rod-curve to long rod and long rod-curve among the isolates (Fig. 1A–F). Although all colonies were opaque on agar, pigmentation varied (white, red, yellow to orange, yellow, purple to pink, or pink to purple). All isolates except SS10D-Y-Pr were able to grow with 0.1% (v/v) methanol. Except Stu1B-Pr, Stu5B-P-Pr, and Stu20C-Re, all isolates were able to grow at 37°C on NMS medium; however, none of the MOB isolates could grow at 45°C.

### Phylogenetic characteristics of the MOB isolates

**Fig. 2:**
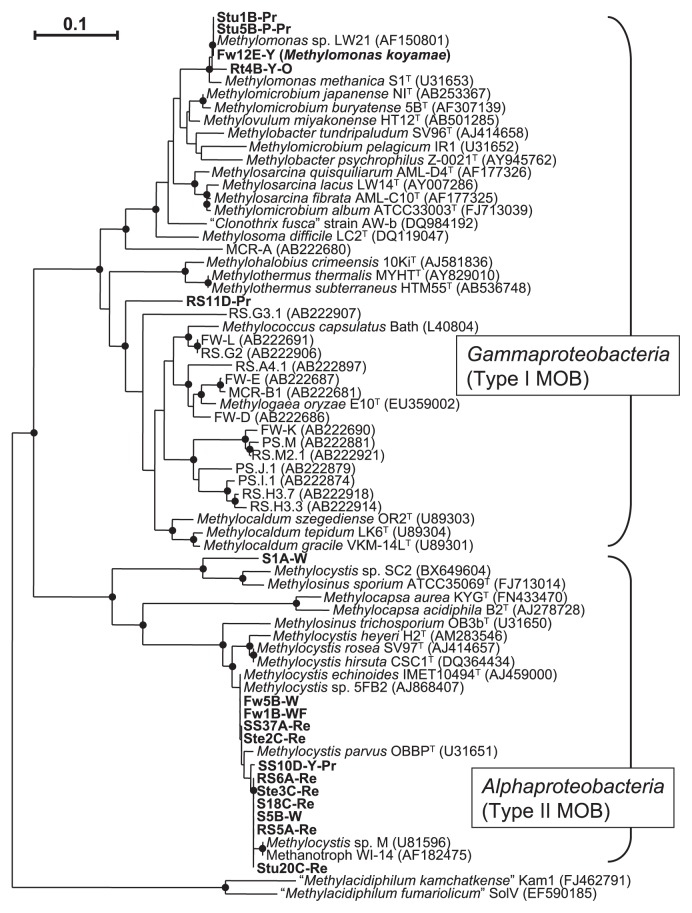
Phylogenetic analysis of the deduced amino acid sequences encoded by *pmoA* genes showing the relationships between the isolates/cultures and other methane-oxidizing bacteria. Bar = 0.1 substitutions per nucleotide sequence position. Bootstrap values more than 50% are shown as closed circles at the branch point. “*Methylacidiphilum kamchatkense*” Kam1 and “*Methylacidiphium fumariolicum*” SolV were used as an outgroup. Accession numbers of reference sequences are shown in parentheses. Deduced amino acid sequences encoded by *pmoA* gene analysis derived from the same paddy field are included. PS, paddy soil (32); RS, rice straw (33); MCR, microcrustaceans in floodwater (43); FW, floodwater (Shibagaki-Shimizu *et al.*, unpublished results).

Sequence analysis of PCR amplification of nearly the full sequence of 16S rRNA genes (1,300–1,400 bp) of MOB isolates was carried out (Table 2). Isolates Stu20C-Re, Ste3C-Re, RS5A-Re, SS37A-Re, SS10D-Y-Pr, and Fw1B-WF were most closely related to *Methylocystis* sp. EB-1 with 97.4–99.7% similarity, while RS6A-Re and S18C-Re, and S5B-W showed 99.7–99.9% and 98.6% similarity to *Methylocystis* sp. 18-2 and *Methylocystis* sp. IMET 10484, respectively. The closest species of these isolates was *Methylocystis parvus*, which showed 96.4–98.8% similarity to type strain OBBP^T^, indicating that the isolates belonged to the genus *Methylocystis* (type II MOB). Four isolates, Fw12E-Y, RS11D-Pr, Stu1B-Pr, and Stu5B-P-Pr, were closely related to uncultured bacterium clone Er-MS-95 (96.1%), uncultured bacterium clone JMYB36-91 (97.8%), *Methylomonas* sp. KSPIII (100%), and *Methylomonas* sp. KSPIII (100%), respectively. The closest species of Fw12E-Y and Stu1B-Pr together with Stu5B-P-Pr were *Methylomonas scandinavica* and *Methylomonas methanica* with 94.9% and 98.3% similarity to type strains R5^T^ and S1^T^, respectively, indicating that the isolates belonged to genus *Methylomonas* (type I MOB). Isolate RS11D-Pr was most closely related to *Methylocaldum szegediense* OR2^T^ (93.1%) (type I MOB) in the known species. To confirm the results from 16S rDNA sequences analysis, a phylogenetic tree was constructed based on the alignment of the deduced amino acid residues (165 amino acid residues) from the *pmoA* gene (Fig. 2). In addition to the 13 MOB isolates, four highly purified MOB cultures (Fw5B-W, S1A-W, Rt4B-Y-O, and Ste2C-Re), which showed good growth and CH_4_ consumption on NMS medium, but contained a few non-MOB like cells, which were observed microscopically or by cultivation in the purity examination described above, were included in the tree. The tree supported the phylogenetic placements of 12 isolates/cultures within the *Alphaproteobacteria* (type II MOB), and five within the *Gammaproteobacteria* (type I MOB). Among the type I MOB isolates/cultures, Fw12E-Y, Stu1B-Pr, Stu5B-P-Pr, and Rt4B-Y-O were most closely related to *Methylomonas* clade, while RS11D-Pr was distantly related to *Methylocaldum-Methylococcus-Methylogaea* cluster. Type II MOB isolates/cultures except for S1A-W were found closely related to *Methylocystis* (*Methylocystis parvus* and *Methylocystis echinoides*) clade, whereas S1A-W belonged to a distant cluster including *Methylosinus sporium*. Deduced amino acid sequences encoded by the *pmoA* gene derived from the same paddy field, *i.e.* paddy soil (32), rice straw (33), microcrustaceans in floodwater (43), and floodwater (Shibagaki-Shimizu *et al.*, unpublished results) were included in the tree in Fig. 2. Only RS11D-Pr showed relationships to *pmoA* sequences of the clones.

### Morphological observation of MOB isolates by TEM and FISH

**Fig. 3:**
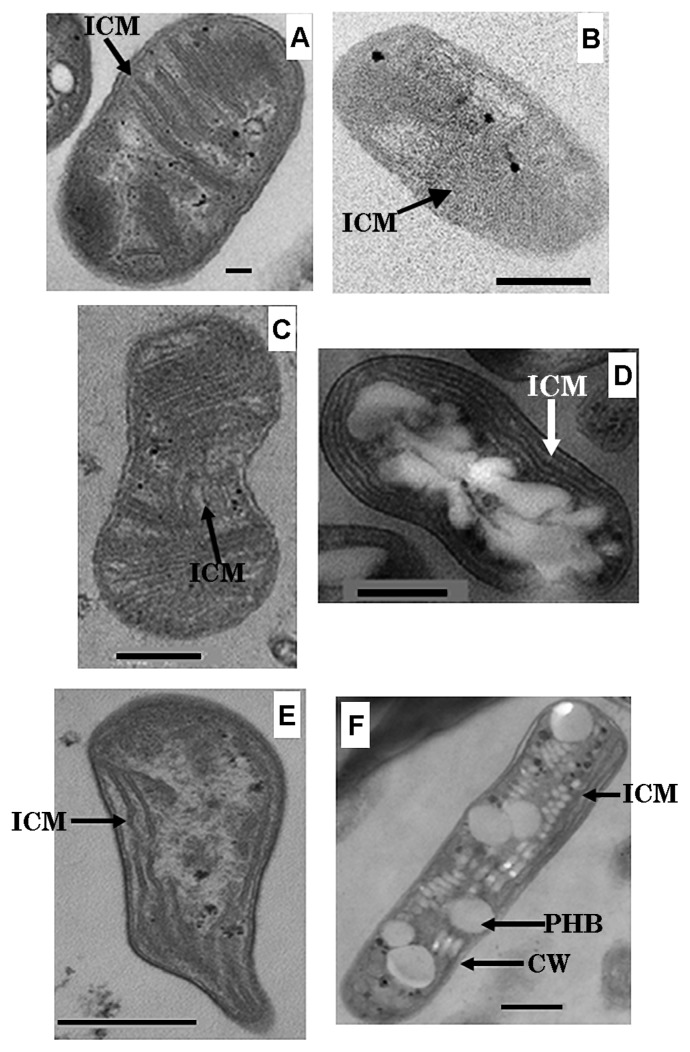
Transmission electron micrograph of thin section of type I-related methane-oxidizing bacterial isolates, (A) Stu1B-Pr, (B) Stu5B-P-Pr, and (C) Rt4B-Y-O, and type II-related methane-oxidizing bacterial isolates, (D) Fw5B-W, (E) S18C-Re, and (F) Rs6A-Re cells. Thin sections were fixed with glutaraldehyde, OsO_4_, and poststained with uranyl acetate and lead citrate. Abbreviations, CW, cell wall; ICM, intracytoplasmic membrane; PHB, poly-β-hydroxybutyrate. Bar=0.1 μm for (A) and 0.5 μm for (B)–(F).

Transmission electron micrograph of ultrathin sections of bacterial cells from the MOB isolates was performed. Isolates Fw12E-Y, Stu1B-Pr, and Stu5B-P-Pr showed a typical internal cytoplasmic membrane (ICM) structure of type I MOB, while the ICM structures of isolates S18C-Re and RS6A-Re were of type II MOB (Fig. 3). The highly purified MOB cultures, Fw5B-W, S1A-W (data not shown), and Rt4B-Y-O contained type I and type II MOB cells with the respective ICM structures (Fig. 3). These results showed good agreement with the phylogenetic placements of the MOB isolates, as revealed by 16S rRNA/*pmoA* gene sequence analysis.

**Fig. 4:**
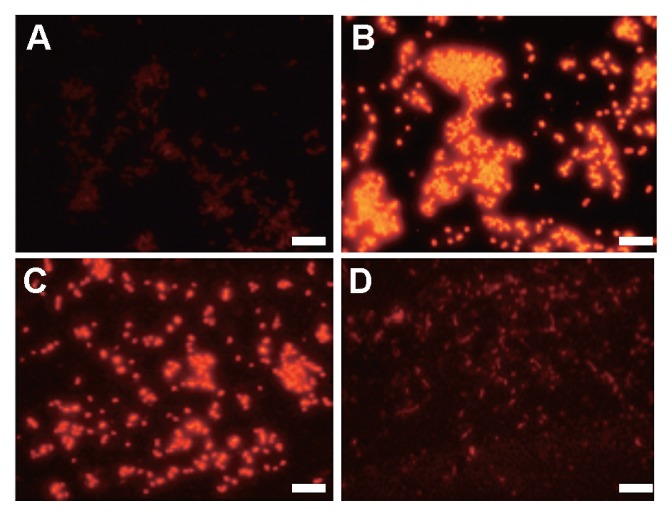
Fluorescence micrographs of methane-oxidizing bacterial isolates Stu1B-Pr [(A) and (B)] and RS6A-Re [(C) and (D)] cells (1 μL) hybridized with specific group probes, Mα450 [(A) and (C)] for type II MOB and Mγ84+Mγ705 [(B) and (D)] for type I MOB. Bar=5.0 μm.

FISH also confirmed the phylogenetic placements of the isolates. Isolate Fw12E-Y, RS11D-Pr, and Stu1B-Pr hybridized with probes Mγ84 and Mγ705, while not hybridizing with probe Mα450. Reversely, the type II MOB isolates, SS37A-Re and RS6A-Re, hybridized well with probe Mα450, and not with the probes for type I MOB. Figure 4 shows the fluorescence micrographs of Stu1B-Pr and RS6A-Re.

### Identification of the MOB isolates

We tried to identify the isolates, focusing mainly on type I-related MOB isolates, in further work and in course of the studies we found very few non-MOB cells in some of the cultures, probably due to accidental contamination during preservation or sub-culturing of the isolates. Finally, strain Fw12E-Y was successfully purified and described as *Methylomonas koyamae* sp. nov. (44). Strain Fw12E-Y represents the second type I MOB isolated from rice paddy field and the first type I MOB strain obtained from a floodwater microsite in a rice paddy field ecosystem to our knowledge.

### Cultivable MOB detected by FISH in MOB enrichment cultures

**Fig. 5:**
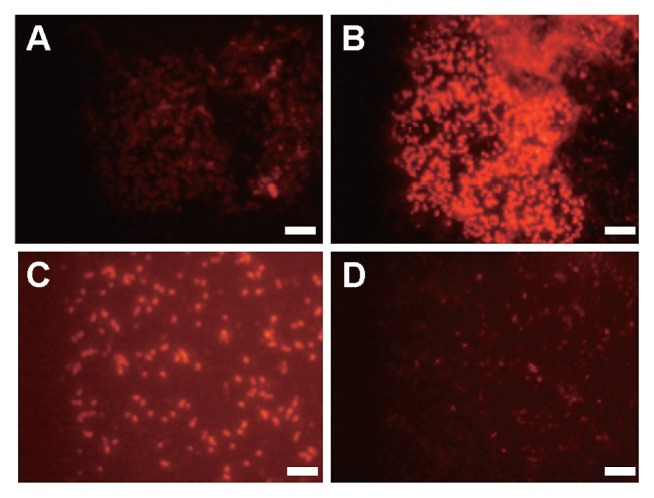
Fluorescence micrographs of bacterial cells extracted from enrichment cultures of floodwater in a rice paddy field [(A) and (B), 10^−1^ dilution under 20% (v/v) CH_4_; (C) and (D), 10^−3^ dilution under 50% (v/v) CH_4_] hybridized with specific group probes, Mα450 [(A) and (C)] for type II MOB and Mγ84 + Mγ705 [(B) and (D)] for type I methane-oxidizing bacteria. Bar=5.0 μm.

Type I and type II MOB were present in the enrichment cultures obtained from all the rice paddy field microsite samples (floodwater, surface soil, bulk soil, rhizosphere soil, root homogenate, and stem of rice plant) collected in 2008. The floodwater enrichment series appeared to contain abundant populations of type I MOB, although certain dilution series seemed to favor type II MOB (Fig. 5).

### Detection of MOB groups in rice paddy field microsites by FISH

**Fig. 6:**
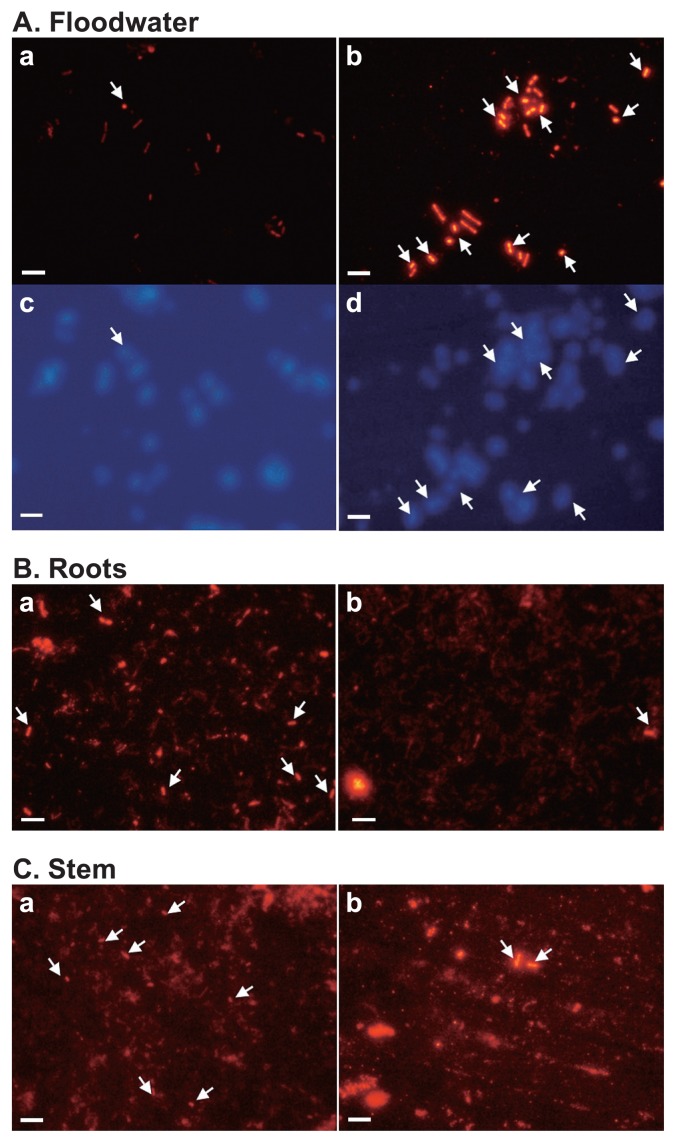
Fluorescence micrographs of bacterial cells (2 μL fixed cells) extracted from rice paddy field microsites, (A) floodwater, (B) roots and (C) stem, hybridized with the specific probes for methane-oxidizing bacterial groups, Mα450 (a) for type II and Mγ84+Mγ705 (b) for type I, and DAPI staining of the same field [(c) and (d), respectively, for (A)]. Arrows show methane-oxidizing bacterial cells hybridized with the probes. Bar=5.0 μm.

Type I and type II MOB were found in all the rice paddy field microsite samples (floodwater, surface soil, bulk soil, rhizosphere soil, root homogenate, and stem of rice plant) collected in 2008, irrespective of the pitfalls of FISH with soil-related samples (1). Type I MOB were found to be abundant in the floodwater, while type II MOB appeared to be more abundant in root and stem microsites of the paddy field in the sampling period (Fig. 6). The ratios of cells of type I MOB to those of type II MOB were about 22, 0.36, and 0.25 for floodwater, root homogenate, and stem of rice plant, respectively. Although accurate counting of bacterial populations revealed by FISH was difficult, especially from soil-related microsite samples (surface soil, bulk soil, rhizosphere soil), due to the autofluorescence of soil particles as reported in several studies (1, 6, 7, 48, 55), the results confirmed the relative abundance of both type I and type II MOB in all the microsites studied, as also indicated by Eller *et al.*(19).

## Discussion

Although the presence of both types I and II MOB in rice paddy field compartments has been reported by many studies (18, 20, 21, 29, 31–33, 35, 38, 40, 50), only two type I strains (*Methylogaea oryzae* [21, 23] and *Methylomonas koyamae* [strain Fw12E-Y; 44]) have been isolated from this environment so far. What could be the limitations of a culture-dependent technique in isolating type I MOB from rice paddy fields? Some studies underlined that methane pressure or shaking during culture incubation in liquid media could favor the growth of type I or type II MOB in the isolation process in addition to varying the culture media (4, 12, 25, 27, 31, 60). Five kinds of enrichment procedures under various culture conditions, such as the concentration of CH_4_, temperature, and shaking or static were used in the present study. While type II MOB were isolated by enrichments AD, type I MOB (Fw12E-Y, RS11D-Pr, Stu1B-Pr, Stu5B-P-Pr and Rt4B-Y-O) were isolated/cultivated by enrichments B, D and E, and in addition we obtained an enrichment culture (SS19A-Pr) containing type I MOB based on the partial 16S rRNA gene sequence from surface soil by enrichment A (data not shown). These findings indicate that culture conditions in the isolation process may have slight influences on the growth preference of type I or type II MOB in the enrichment culture. One reason could also be the difficulty of separating type I MOB cells from non-MOB contaminants, as reported by Ferrando and Tarlera (21) and Geymonat *et al.*(23), and repeated transfer of colonies on agar slants or plates to liquid media and subsequent sub-culturing may lead to the loss of type I MOB cells. Type I MOB have been found to be preferentially grazed by protozoa in rice paddy fields (41, 42), which may also support the difficulty in isolating type I from this ecosystem. As underlined by Leadbetter (36), this enormous gap between the diversity of cultivated methanotrophs and the diversity of MOB in the rice paddy field ecosystem may partially be linked to our insufficient knowledge or imagination of the chemistry of their extracellular milieu. Therefore, refining and improving the isolation media and conditions may yield more type I MOB and consequently lead to deep insight into the true diversity of cultivable MOB in the rice paddy field ecosystem. To obtain the best picture of the methanotroph community present in the rice paddy field ecosystem, it is suitable to combine both molecular and cultivation techniques with regard to the limitation of both methods (17).

In this study, we combined the FISH method and the cultivation technique to characterize the cultivable methanotroph communities present in seven microsites of the paddy field ecosystem, and we compared our data with those obtained from the paddy field using *pmoA* gene analysis. Floodwater and stump compartments are very poorly studied niches in the rice paddy field ecosystem. The isolates/cultures consisted of 12 type II and five type I MOB. The five type I MOB were issued from floodwater (Fw12E-Y), rhizosphere soil (RS11D-Pr), root (Rt4B-Y-O), and stump (Stu1B-Pr, Stu5B-P-Pr), respectively, and strain Fw12E-Y was found to represent a new species in the genus *Methylomonas*(44), the first type I MOB isolated from floodwater in a rice paddy field. Type II MOB were isolated from almost all microsites except the root and were affiliated with *pmoA* sequences of the *Methylocystis* cluster or *Methylosinus sporium* cluster; the findings were in accordance with previous studies using a cultivation technique (16, 24, 52, 53). Identification of these isolates/cultures is necessary in future work. The isolation of type I MOB in pure culture proved very difficult. As also reported by Ferrando and Tarlera (21), despite repeated streaking on different solid media, phase-contrast microscopic examination of cells from well-isolated colonies revealed the presence of contaminants with typical morphological features of *Hyphomicrobium*-like cells. In agreement with these authors, repeated transfer of colonies on highly purified agar slants to liquid medium, and subsequent subculturing may allow the isolation of type I methanotrophs from the highest dilution series.

The phylogenetic placement of MOB isolates was in agreement with studies conducted on floodwater, bulk soil, rice straw, and microcrustaceans in the rice paddy field based on *pmoA* analysis (32, 33, 43, Shibagaki-Shimizu *et al.* unpublished results). Almost all the type II isolates were associated with *Methylocystis parvus* or *Methylocystis echinoides* (Fig. 2); however, S1A-W was related to *Methylosinus sporium*. RS11D-Pr (type I-like MOB isolate) was distantly related to clones from floodwater (FW), rice straw (RS), and microcrustaceans (MCR), together with a novel species *Methanogaea oryzae* from the soil-water interface in a flooded rice field in Uruguay (21, 23) (Fig. 2). We also obtained an enrichment culture (Fw10D-Pr) showing typical morphological features the same as RS11D-Pr (Fig. 1) from floodwater. Isolates/cultures of Fw12E-Y, Stu1B-Pr, Stu5B-P-Pr, and Rt4B-Y-O were associated with the *Methylomonas* cluster and did not show any close relationships to *pmoA* sequences of the clones. That might be related to the different paddy field conditions during both studies; however, the relative abundance found for *Methylomonas*-related MOB isolates in stump and floodwater microsites seemed in agreement with the results of Ferrando and Tarlera (21). Using *pmoA*-base clones libraries analysis, they found for one group (group 3), 44.2% and 4.5% of clone species related to *Methylomonas* species at the soil-water-interface (SWI) and rhizosphere soil, respectively. The SWI microsite was related to a similar environment to the floodwater and stump microsites in our study from which we obtained the *Methylomonas* cultures.

Using the FISH technique, type I and type II MOB were found in all the microsites of the paddy field as well in the enrichment cultures. The presence of type I methanotrophs in these cultures may anticipate more type I MOB in pure cultures from the rice paddy field ecosystem. The relative abundance of type I and type II MOB found in different microsites of the rice field overall resembled the results in a rice microcosm system obtained by Eller and Frenzel (18) with the same group-specific probes; they reported that type II MOB predominated over type I MOB in bulk soil, rhizosphere and rhizoplane, but type I MOB also occurred in the compartments, especially at a higher relative proportion in the rhizoplane. In contrast, the proportion of type II MOB seemed to be higher in root homogenate in the present study. One principal reason may be the difficulty in counting cells, with another reason being the differences in paddy fields conditions.

For this study we focused on seven compartments of the rice paddy field ecosystem. Using combined cultivation and FISH techniques, the presence of both type I and type II methanotrophs was revealed. We successfully isolated strain Fw12E-Y from floodwater and obtained four type I MOB cultures from the stump, root and rhizosphere soil, respectively. Although their presence in these microsites has been shown in rice paddy fields using molecular approaches, the isolation of type I MOB has not been reported to our knowledge from these microsites of a rice paddy field. As Leadbetter (36) and Donachie *et al.*(17) underlined, in agreement with Hengstmann *et al.*(28) and Oremland *et al.*(45), by implementing comprehensive strategies that include culture-dependent techniques and molecular approaches, we can identify the full extent of microbial diversity in a given environment. From the present study, we elucidated the extent of the diversity of cultivable methanotrophs in a rice paddy field ecosystem including type I MOB, for which only one isolation has been recently reported so far (23).

## Figures and Tables

**Table 1:** Properties of methane-oxidizing bacterial isolates from rice paddy field microsites

Isolate^1^	Cell morphology^2^	Motility	Exospore formation	Gram stain	Colony aspect^3^	Growth on 0.1% methanol	Growth at 37°C	Growth at 45°C
Fw12E-Y	SR	+	−	−	Y-Or, O	+	+	−
Fw1B-WF	LRC	+	−	−	W, O, F	+	+	−
SS10D-Y-Pr	SR	+	−	−	Y, O, G	−	+	−
SS37A-Re	LR	+	−	−	Re, O	+	+	−
S5B-W	SR	+	−	−	W, O	+	+	−
S18C-Re	R	+	−	−	Re, O	+	+	−
RS5A-Re	R	+	−	−	Re, O	+	+	−
RS6A-Re	R	+	−	−	Re, O	+	+	−
RS11D-Pr	RC	+	−	−	Pr-P, O	+	+	−
Ste3C-Re	R	+	−	−	Re, O	+	+	−
Stu1B-Pr	LR	+	−	−	P-Pr, O, G	+	−	−
Stu5B-P-Pr	R	+	−	−	P-Pr, O, G	+	−	−
Stu20C-Re	R	+	−	−	Re, O	+	−	−

1 Name of isolates was designated with the source, isolate number, enrichment method, and pigment: Fw, floodwater; SS, surface soil; S, bulk soil; RS, rhizosphere soil; Ste, stem; Stu, stump. A-E, enrichment methods. Y, yellow; W, white; Y-Pr, yellow to purple; Re, red; Pr, purple; P-Pr, pink to purple; F, filamentous. Isolates were cultivated at 30°C.

2 R, rod; SR, short rod; RC, rod curved; SRC, short rod curved; LR, long rod; LRC, long rod curved.

3 W, white; Y, yellow; Y-Or, yellow to orange; Re, red; P-Pr, pink to purple; Pr-P, purple to pink. O, opaque; F, filamentous; G, gelatinous.

**Table 2:** Closest species of methane-oxidizing bacterial isolates from rice paddy field microsites based on 16S rRNA gene sequence

MOB isolate	Alignment (Similarity %)	Closest species (Strain number in culture collection^1^)	Taxonomic description	Accession number
Fw12E-Y	1271/1339 (94.9)	*Methylomonas scandinavica* R5^T^ (VKM B-2140)	*Gammaproteobacteria* (type I)	AJ131369
Fw1B-WF	1385/1402 (98.8)	*Methylocystis parvus* OBBP^T^ (NCIMB 11129)	*Alphaproteobacteria* (type II)	Y18945
SS10D-Y-Pr	1365/1390 (98.2)	*Methylocystis parvus* OBBP^T^ (NCIMB 11129)	*Alphaproteobacteria* (type II)	Y18945
SS37A-Re	1355/1405 (96.4)	*Methylocystis parvus* OBBP^T^ (NCIMB 11129)	*Alphaproteobacteria* (type II)	Y18945
S5B-W	1365/1395 (97.8)	*Methylocystis parvus* OBBP^T^ (NCIMB 11129)	*Alphaproteobacteria* (type II)	Y18945
S18C-Re	1383/1404 (98.5)	*Methylocystis parvus* OBBP^T^ (NCIMB 11129)	*Alphaproteobacteria* (type II)	Y18945
RS5A-Re	1353/1394 (97.1)	*Methylocystis parvus* OBBP^T^ (NCIMB 11129)	*Alphaproteobacteria* (type II)	Y18945
RS6A-Re	1389/1406 (98.8)	*Methylocystis parvus* OBBP^T^ (NCIMB 11129)	*Alphaproteobacteria* (type II)	Y18945
RS11D-Pr	1351/1451 (93.1)	*Methylocaldum szegediense* OR2^T^ (NCIMB 11912)	*Gammaproteobacteria* (type I)	U89300
Ste3C-Re	1382/1405 (98.4)	*Methylocystis parvus* OBBP^T^ (NCIMB 11129)	*Alphaproteobacteria* (type II)	Y18945
Stu1B-Pr	1428/1452 (98.3)	*Methylomonas methanica* S1^T^ (NCIMB 11130)	*Gammaproteobacteria* (type I)	AF304196
Stu5B-P-Pr	1428/1452 (98.3)	*Methylomonas methanica* S1^T^ (NCIMB 11130)	*Gammaproteobacteria* (type I)	AF304196
Stu20C-Re	1389/1408 (98.7)	*Methylocystis parvus* OBBP^T^ (NCIMB 11129)	*Alphaproteobacteria* (type II)	Y18945

1 NCIMB, The National Collections of Industrial and Marine Bacteria; VKM, All-Russian Collection of Microorganisms.
